# Molecular Characterization of a Rare Case of Monozygotic Dichorionic Diamniotic Twin Pregnancy after Single Blastocyst Transfer in Preimplantation Genetic Testing (PGT)

**DOI:** 10.3390/ijms231810835

**Published:** 2022-09-16

**Authors:** Sophie Brouillet, Sandie Mereuze, Noémie Ranisavljevic, Claire Chauveau, Samir Hamamah, Julie Cattin, Camille Verebi, Christelle Cabrol, Aliya Ishmukhametova, Anne Girardet, Tal Anahory, Marjolaine Willems

**Affiliations:** 1 DEFE, University of Montpellier, INSERM, Montpellier, France; 2 Biologie de la Reproduction/DPI et CECOS, Département de Biologie de la Reproduction, CHU of Montpellier, Montpellier, France; 3 Laboratory of Molecular Genetics, CHU of Montpellier, Montpellier, France; 4 Department of Reproductive Medicine, CHU of Montpellier, Montpellier, France; 5 Department of Medical Genetics, CHU of Montpellier, Montpellier, France; 6 Service de Gynécologie-Obstétrique, CHU Jean Minjoz, Besançon, France; 7 Service de Médecine Génomique, Maladies de Système et d’Organe, Fédération de Génétique et de Médecine Génomique, DMU BioPhyGen, APHP Centre—Université Paris Cité, Hôpital Cochin, Paris, France; 8 Centre de Génétique Humaine, CHU Jean-Minjoz, Besançon, France; 9 PhyMedExp, CHU of Montpellier, University of Montpellier, INSERM, CNRS, Montpellier, France; 10 Reference Centre AD SOOR, AnDDI-RARE, INSERM U1298, INM, Department of Medical Genetics, Arnaud de Villeneuve Hospital and University of Montpellier, Montpellier, France

**Keywords:** preimplantation genetic testing, blastocyst, pregnancy, monozygotic, twin, dichorionic, diamniotic

## Abstract

Preimplantation genetic testing (PGT) is widely used to select unaffected embryos, increasing the odds of having a healthy baby. During the last few decades, it was accepted that monozygotic dichorionic diamniotic twin pregnancies occurred from the embryo splitting before Day 3 postfertilization according to Corner’s dogma. Hence, the occurrence of a dichorionic diamniotic twin pregnancy after a single blastocyst transfer was considered a dizygotic pregnancy resulting from blastocyst transfer and concurrent natural fertilization. In our study, we have provided for the first time molecular proof that a single blastocyst transfer can result in a monozygotic dichorionic diamniotic twin pregnancy, invalidating Corner’s dogma. In this case, we recommend systematically assessing the genetic status of dichorionic twins after single blastocyst transfer using prenatal diagnosis to exclude the risk from a potential concurrent spontaneous pregnancy and to ensure that both fetuses are unaffected. To achieve this goal, we have developed here an innovative noninvasive prenatal diagnosis by exclusion of paternal variants with droplet digital PCR, maximizing the reliability of genetic diagnosis. Further multicentric prospective studies using genetic testing are now required to establish the rate of blastocyst splitting leading to dichorionic pregnancy in PGT and to identify the risk factors.

## 1. Introduction

Preimplantation genetic testing (PGT) is widely used on human embryos obtained by in vitro fertilization (IVF) to detect monogenic defects (PGT-M), chromosomal structural rearrangements (PGT-SR), or aneuploidy (PGT-A). The aim of PGT is to select unaffected embryos for uterine transfer, increasing the odds of having a healthy baby. To date, there is still wide variation in PGT policy between countries, with stricter regulations in European countries compared to North America [1,2,3]. Compared to natural pregnancy, the likelihood of multifetal pregnancy is higher in PGT, mainly due to the transfer of more than one embryo into the uterus [4]. Interestingly, the incidence of monozygotic twins is also increased in IVF compared with spontaneous conception (from 0 to 13.2% [5] versus 0.4% [6,7], respectively), probably due to embryo micromanipulation (e.g., intracytoplasmic sperm injection, assisted hatching, extended culture, embryo biopsy) during in vitro preimplantation embryo development [8,9].

**Figure 1:**
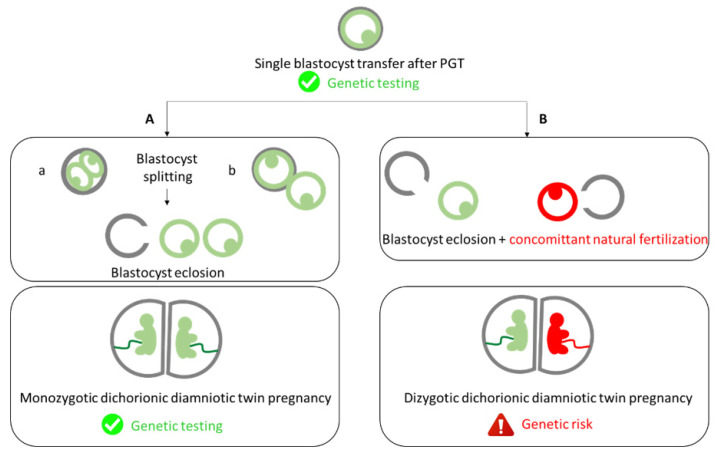
Schematic representation of the embryonic mechanisms that can lead to dichorionic diamniotic twin pregnancy. (**A**) Blastocyst splitting (a) before or (b) after (the “8” shape) its transfer leading to a monozygotic dichorionic diamniotic twin pregnancy. (**B**) Blastocyst transfer and concurrent spontaneous pregnancy leading to a dizygotic dichorionic diamniotic twin pregnancy.

Currently, there are controversies concerning the origin of monozygotic twin pregnancy after single embryo transfer (Figure 1) [10]. The development model of monozygotic twins described by Corner in 1955 has become accepted as the golden rule [11]. During the last few decades, it was accepted that monozygotic dichorionic diamniotic twin pregnancies occurred from the embryo splitting before Day 3 postfertilization, monochorionic diamniotic twin pregnancies between 4–8 days postfertilization, and monochorionic monoamniotic twin pregnancies between 9–12 days [11,12]. Based on this dogma, it was believed that single blastocyst transfer could only result in monochorionic twin pregnancy. Therefore, the occurrence of a dichorionic diamniotic twin pregnancy after a single blastocyst transfer was considered a dizygotic pregnancy resulting from blastocyst transfer and concurrent natural fertilization. In 2013, Kyono and colleagues proved the monozygosity of a dichorionic diamniotic twin pregnancy after the transfer of a single blastocyst using DNA fingerprinting (short tandem repeats) [13], questioning the original dogma, and suggesting that a blastocyst can spontaneously split in half in the uterus. Nevertheless, genetic proof that the pregnancy came from the transferred blastocyst (and not from a concurrent natural fertilization) is still lacking.

In the last decade, reports of dichorionic diamniotic twin pregnancies after a single embryo transfer have increased [8,14]. Dichorionic diamniotic twin pregnancies would occur in 25–30% of twin pregnancies after single embryo transfer (versus 70–75% of monochorionic diamniotic pregnancies and 1–2% of monochorionic monoamniotic pregnancies), which seem consistent with the data of natural conceptions [15]. However, these data should be considered with caution, as evidence supporting chorionicity was absent or incomplete in these previous studies. Indeed, first trimester ultrasound scans showing a lambda sign and proving the chorionicity were missing. Moreover, the assessment of embryo zygosity of dichorionic diamniotic twin pregnancies using genetic testing has rarely been performed, leading to falsely considering these pregnancies as dizygotic [16]. Consequently, the monozygotic twinning rate was highly underestimated. Recently, Konno et al. (2020) used genetic testing to estimate that half of dichorionic diamniotic twin pregnancies after single embryo transfer are monozygotic [17].

In PGT, the identification of a dichorionic diamniotic twin pregnancy after single embryo transfer raises important questions about the zygosity, and therefore about the genetic risk of the pregnancy. Indeed, it is estimated that 10–35% of twins born after single embryo transfers in IVF result from a concurrent natural conception (Figure 1) [13,18].

In PGT, this could lead to the implantation of undiagnosed embryos and potentially to the birth of affected children. Thus, it is essential to better evaluate the genetic risk in dichorionic diamniotic twin pregnancy after single embryo transfer and create strategies to manage these pregnancies.

To date, only a few studies have used genetic testing (and not fetal sex) to establish monozygosity in dichorionic diamniotic twin pregnancy after single embryo transfer [13,17,19]. In addition, no publication has demonstrated the genetic concordance between monozygotic dichorionic twins and the transferred blastocyst, which leaves doubt about the origin of the pregnancy (i.e., embryo splitting or concurrent spontaneous pregnancy). In this study, we diagnosed a dichorionic diamniotic twin pregnancy after a single embryo transfer in PGT-M for spondyloepiphyseal dysplasia. Our objectives were to assess the genetic status of the twins using noninvasive prenatal diagnosis (to minimize the risk of miscarriage) by paternal variant exclusion, to reassure the couple about the absence of the *COL2A1* pathogenic variant in both fetuses, and to study the genetic concordance between the twins and the transferred blastocyst to evaluate the accuracy of Corner’s dogma [11].

## 2. Results

A couple requesting PGT-M for autosomal dominant spondyloepiphyseal dysplasia linked to a pathogenic variation of the COL2A1 gene in the male partner was referred to our center.

Table 1 displays the clinical and biological parameters associated with the PGT-M cycle.

- Controlled ovarian stimulation

A total of 17 cumulus-oocyte complexes were collected during ovarian puncture. No liquid in the peritoneal cavity or follicles of more than 10 mm in diameter were detected at the end of the procedure.

- Fertilization, embryo culture, and embryo biopsy

Sperm parameters were above the fifth percentile according to the Manual for the Laboratory Examination and Processing of Human Semen, WHO (2021, sixth edition). A total of 17 mature oocytes (metaphase II) were fertilized using intracytoplasmic sperm injection (100% of mature oocytes). A total of 11 zygotes were observed on Day 1. On Day 3, 12 embryos were obtained, and 10 embryos were biopsied.

- PGT-M

**Figure 2:**
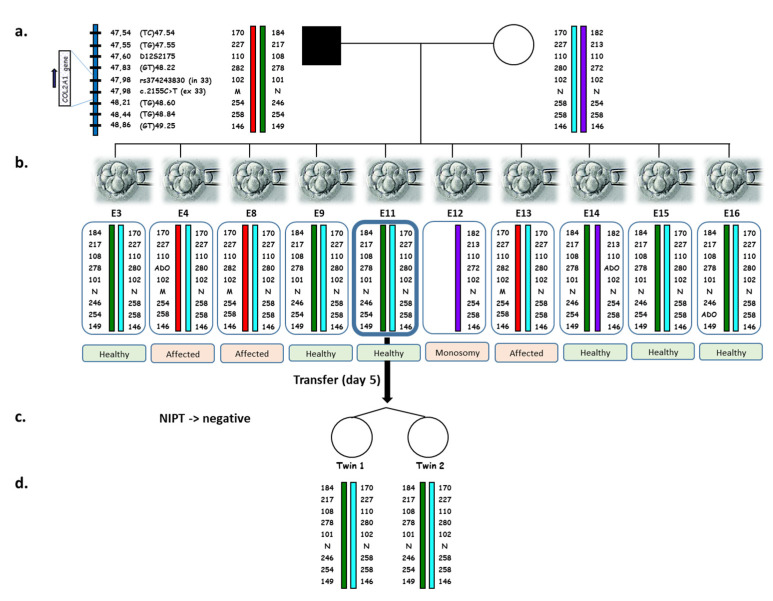
Pedigree and haplotypes at the *COL2A1* locus obtained for the couple, PGT embryos, and twins. (**a**) Alleles from top to bottom correspond to the 7 extragenic informative STRs and the intragenic SNP, encompassing the *COL2A1* c.2155C > T site, from centromere to telomere. Genomic locations (GRCh38/hg38) are indicated on the left of each marker. Allele length (in base pairs) is indicated for each marker for the couple, PGT embryos, and twin girls. M: mutant allele (c.2155T); N: normal allele (c.2155C). (**b**) PGT results obtained on one single blastomere from the 10 biopsied embryos on Day 3. ADO, Allele Drop Out. Embryo 11 was transferred on Day 5. (**c**) Noninvasive prenatal testing was performed at 12+3 WG. The results did not reveal the presence of the paternal *COL2A1* variant. (**d**) Postnatal genetic testing of the twins at the *COL2A1* locus showing identical results compared to the transferred embryo.

The PGT-M genetic results are shown in Figure 2.

All of the blastomeres successfully amplified for the nine DNA sequences, and only two allele drop out (ADO, the random amplification failure of one of the two alleles in a heterozygous sample) were detected for the (GT)48.22 marker—one of paternal origin and one of maternal origin—without affecting the reliability of PGT-M. A total of six unaffected embryos were identified on Day 4.

- Embryo transfer

A single blastocyst was transferred on the fifth day after ovarian puncture (4AA stage according to [20]). The selection of this embryo was made after the evaluation of its morphological criteria by two practitioners. Both agreed on the presence of a unique inner cell mass and a unique trophectoderm within the zona pellucida. Intra- and inter-observer quality controls were carried out four times a year in the laboratory by the internal and external quality referents. The two practitioners fully validated these quality controls. The endometrial thickness was 12 mm. Three supernumerary unaffected blastocysts were cryopreserved on Day 5 (*n* = 2) and on Day 6 (*n* = 1).

- Pregnancy

**Figure 3:**
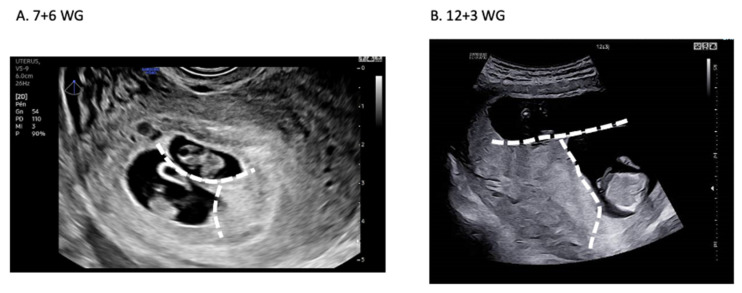
Ultrasound assessment of dichorionic diamniotic twin pregnancy. (**A**) First ultrasound scan at 7+6 WG showing two amniotic gestational sacs with a “lambda” sign referring to the triangular appearance of the chorion (dotted white line). (**B**) Confirmation of the dichorionic diamniotic twin pregnancy at 12+3 WG.

A positive pregnancy test was obtained 12 days following embryo transfer (β-hCG 319 UI/l), confirmed 48 h later (464 UI/l). The first ultrasound scan performed at 7+6 weeks of gestation (WG) showed two amniotic gestational sacs with a thick septum membrane (Figure 3A).

A “lambda” sign referring to the triangular appearance of the chorion indicates a dichorionic diamniotic twin pregnancy (Figure 3A) [21]. Both fetal heartbeats were detected, and the height of each fetus was estimated (Twin #1: 13 mm and Twin #2: 15.1 mm). The dichorionic diamniotic twin pregnancy was confirmed with a second ultrasound at 12+3 WG (Figure 3B), questioning the zygosity and the reliability of the genetic diagnosis.

- Noninvasive prenatal diagnosis

**Figure 4:**
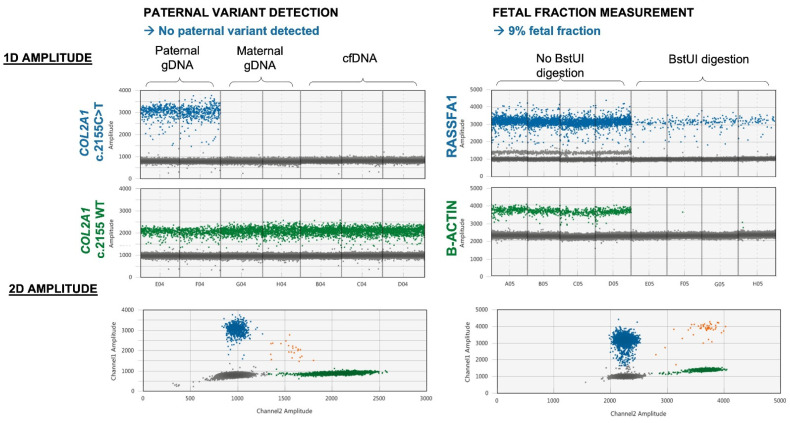
Noninvasive prenatal diagnosis by paternal variant exclusion: results of droplet digital PCR amplification. The presence of fetal DNA in maternal plasma was confirmed through the detection of RASSF1A-positive droplets after BstUI digestion. Parental genomic DNA (gDNA) was tested concomitantly with maternal plasma cell-free DNA (cfDNA) as positive and negative controls.

A specific assay for the paternal pathogenic variant was designed at 9+6 WG. Maternal blood sampling for noninvasive prenatal diagnosis was performed at 12+3 WG. The fetal fraction—indistinguishably associated with the presence of twins—was evaluated at 9%, and no paternal variant was detected in maternal plasma cell-free DNA (Figure 4).

Therefore, we concluded that no fetus carried the paternal variant and reassured the couple.

- Genetic testing of twins

**Figure 5:**
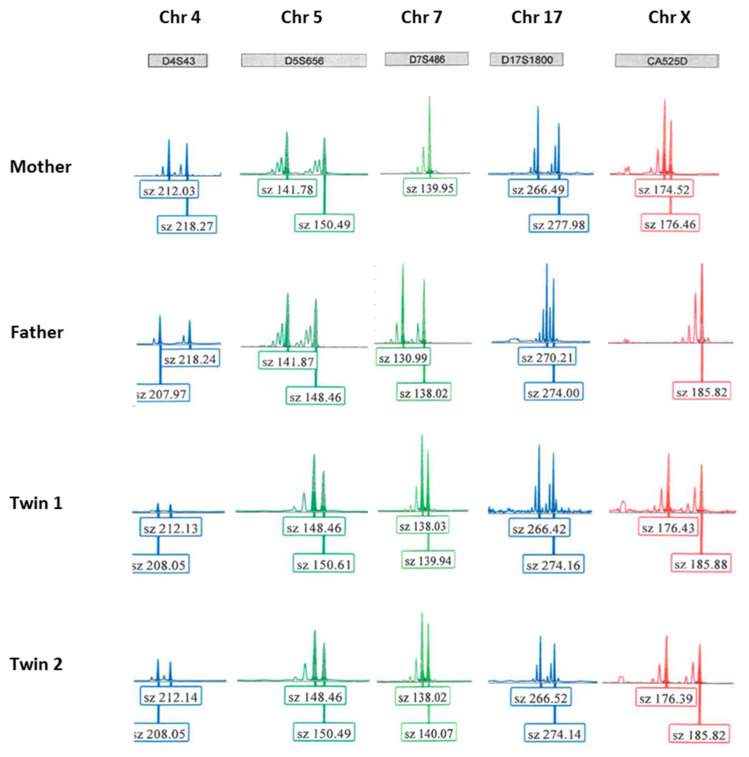
Example of electropherograms from the couple and the baby twins. The results obtained for 5 informative STR markers (D4S43, D5S656, D7S486, D17S1800, and CA525D, located on chromosomes 4, 5, 7, 17, and X, respectively) are shown. The sizes of the marker alleles (in bp) are indicated below the electropherograms. Sz: size.

The birth occurred at 36 WG. The patient underwent labor induction by balloon catheter and oxytocin for cholestasis of pregnancy with hepatic cytolysis and severe pruritus. Healthy female twins weighed 2400 g and 2090 g. Buccal swab samples were collected from the twin babies at 6 weeks old, and DNA was analyzed. The determination of zygosity was made by STR analysis using a total of 64 STR markers distributed on chromosomes 4, 5, 7, 17, and X. The genotypes of the twins were identical at all informative markers (Figure 5), making monozygosity highly likely.

The calculated probability for the twins to be monozygotic twins was 99.976%. We performed multiplex PCR amplification for the COL2A1 gene (eight polymorphic DNA sequences) and compared the results to those of the transferred embryo. The results were identical between the twins, as well as between the twins and the Day 5 transferred blastocyst.

## 3. Discussion

In this study, we demonstrate for the first time using genetic testing that a single blastocyst transfer can result in a monozygotic dichorionic diamniotic twin pregnancy, which invalidates Corner’s dogma claiming that blastocyst transfer could only result in a monochorionic twin pregnancy [11]. Whenever possible in PGT, it is useful to evaluate through genetic testing the concordance between the twins and the single transferred embryo to identify the origin of the pregnancy. This contributes to a better understanding of, and improved safety of, the twin pregnancy. Since dichorionic pregnancy can also result from concurrent natural fertilization in a single embryo transfer cycle, it is necessary to assess the genetic status of dichorionic twins using prenatal diagnosis to ensure that both fetuses are unaffected. To achieve this goal, we have developed here an innovative noninvasive prenatal diagnosis by exclusion of paternal variants with droplet digital PCR, maximizing the reliability of genetic diagnosis.

Most noninvasive exclusion of paternal variant reports published to date rely on massively parallel sequencing (MPS) (as recently published for skeletal dysplasia [22]). With an experimental workflow of a few hours, noninvasive prenatal diagnosis by paternal variant exclusion with droplet digital PCR appears to be a valuable option considering MPS costs and turnaround time, as sample processing and sequencing take several days. Although a specific assay is needed for each targeted variant in noninvasive prenatal diagnosis with droplet digital PCR, this cautious conception and validation ensures optimal specificity and sensitivity, as no false positives or false negatives have been reported to date [23]. As there is still controversy about a higher risk of miscarriage of amniocentesis in twin pregnancy than in singleton pregnancy [24,25], a reasonable, safe approach is to prioritize noninvasive alternatives whenever they are available.

The first case of monozygotic twin pregnancy associated with IVF was reported in 1984 [26]. Since then, its incidence has continually risen [27]. In recent decades, monozygotic dichorionic twin pregnancies have been systematically considered to be the result of cleaved embryo splitting before inner cell mass differentiation [12]. This dogma was supported by sparse experimental data, mainly obtained from nonhuman embryos [28,29,30,31]. Recently, some authors strongly questioned this dogma [32,33], reporting the spontaneous splitting of blastocysts in vitro (resulting in two half-blastocysts containing both inner cell mass and trophectoderm components within the zona pellucida [34] (Figure 1A(a)) or more often while escaping the zona pellucida after forming the “8” shape (Figure 1A(b)) [35]). However, no data have thus far proven that a blastocyst can divide after its transfer within the uterine cavity into two developing blastocysts, giving rise to monozygotic dichorionic pregnancy.

In this study, the genetic results were in concordance with the clinical data supporting the absence of spontaneous ovulation (i.e., the absence of ovarian follicles > 10 mm after ovarian puncture and absence of liquid into the uterine cavity during embryo transfer) and with protected sexual intercourse reported by the couple during the entire PGT procedure, which undermined the hypothesis of a concurrent natural conception. The strengths of our study include: (1) the assessment of the genetic status of the twins at different periods of their development (fetal and neonatal stages), (2) the evaluation of twin zygosity using molecular genetics analysis (which is the gold standard) and not only by comparing the gender among the fetuses as in most of the studies published to date, and, especially, (3) the evaluation of the genetic concordance between the twins and the transferred embryo, which is an approach that had never been reported thus far in the literature. Moreover, this is the first case reported in the literature of the successful birth of monozygotic dichorionic diamniotic twins after a single-blastocyst transfer in PGT using genetic testing. Dichorionic diamniotic twin pregnancy is a rare event after single embryo transfer (∼2% risk of multiple pregnancies, of which up to 30% can be dichorionic [15,36]), but its occurrence in PGT is associated with increased genetic risks if one of the embryos originates from spontaneous fertilization (which occurs in 10–35% of twins born after single embryo transfers [13,18]). Hence, case reports are necessary to enlarge the literature and improve our understanding of the associated risk factors.

To date, the key factors affecting the prevalence of embryo splitting are still unknown. One could speculate that epigenetic alterations are associated with preimplantation embryo micromanipulation and culture, leading to an increased rate of monozygotic dichorionic pregnancies in IVF. A recent meta-analysis of 40 studies reported that blastocyst transfer, female age below 35 years, conventional IVF, and assisted hatching were significantly associated with an increased monozygotic pregnancy rate after IVF [8]. Interestingly, embryo biopsy, embryo cryopreservation, and oocytes’ donation were not associated with monozygotic pregnancies [8]. Further studies are still required to evaluate the specific risk factors of monozygotic dichorionic pregnancies and their associated epigenetic alterations.

In view of our data and the current literature, we propose the following recommendations to help multidisciplinary teams optimally manage dichorionic pregnancy after single embryo transfer in PGT:

(1) Inform the couple about the possibility that the pregnancy results from the splitting of the transferred embryo (regardless of its day of transfer) or from the occurrence of a spontaneous concurrent pregnancy, which exposes them to have an affected child. After having informed them of this risk, ask them about unprotected sexual intercourse, which could be the origin of a spontaneous pregnancy;
(2) Recommend a prenatal diagnosis to evaluate the genetic status of the fetus. This becomes strongly recommended if there has been potentially fertilizing sexual intercourse or if the fetuses are of different genders. Clearly inform the couple that same-sex twins do not guarantee monozygosity or genetic concordance with the diagnosed embryo. If possible, a noninvasive approach should be proposed to minimize the risk of miscarriage associated with choriocentesis or amniocentesis [37,38];
(3) We propose the evaluation of twin zygosity using genetic testing. If there remains DNA from the biopsied embryo that was subsequently transferred (this may occur if a whole genome amplification technique has been performed before locus-specific amplification), systematically evaluate the genetic concordance between the transferred embryo and the twins. Indeed, the underlying mechanisms leading to blastocyst splitting are still unknown. The increase in the number of case reports will allow us to better understand the risk factors, assuring the safety of dichorionic pregnancy after single embryo transfer in PGT. Moreover, it will also contribute to the improvement of our knowledge of pre- and peri-implantation embryonic development.

## 4. Materials and Methods

### 4.1. Ethics

This study was approved by the Ethics Committee and the Institutional Research Review Board of the University of Montpellier (IRB-MTP_2022_06_202201122). Informed consent was sought and received from the participating couple. The data analysis was carried out according to the principles of the Declaration of Helsinki.

In France, the current legislation strictly regulates PGT (law of Bioethics of 1994 n°94-654). PGT-M and PGT-SR can only be performed for a serious and incurable genetic disorder. PGT-A is currently not allowed in France.

### 4.2. Patients

A nonconsanguineous couple was referred to our IVF unit seeking PGT-M for autosomal dominant spondyloepiphyseal dysplasia in the male partner linked to a pathogenic variation of the *COL2A1* gene. This pathology was segregated in the male partner’s family, in which all of the affected members were heterozygous for a missense mutation (c.2155C>T, p. Arg719Cys) in exon 33 of the *COL2A1* gene (NM_001844), encoding the collagen alpha-1(II) chain [39]. Type II collagen is specific for cartilaginous tissues. It is essential for the normal embryonic development of the skeleton, for linear growth and for the ability of cartilage to resist compressive forces. Several skeletal and ocular disorders were found to be caused by variations in the *COL2A1* gene, including spondyloepiphyseal dysplasia [40]. The patient, his sister, and their father had evolutive spondyloepiphyseal dysplasia, whose first symptoms began before 10 years of age and were characterized by abnormal epiphyses, flattened vertebral bodies, and severe premature osteoarthritis, without short stature, myopia, or deafness. Due to the relatively severe clinical presentation in this family, and the phenotype uncertainty for an affected child of the couple, the Multidisciplinary Center for Prenatal Diagnosis accepted the indication of a PGT-M or a prenatal diagnosis. The couple did not seek a prenatal diagnosis, but they opted for a PGT-M procedure.

### 4.3. PGT Work-Up

The PGT-M relied on both indirect genetic diagnosis using informative markers and direct genetic diagnosis of the causative pathogenic variant. Informativity was defined according to the ESHRE PGT consortium’s good practice recommendations for the detection of monogenic disorders [41]. A total of 17 polymorphic short tandem repeat (STR) markers and 3 single nucleotide polymorphisms (SNPs) were identified in or on each side of the *COL2A1* gene on chromosome 12 to determine the haplotypes linked to the mutant and normal genes in the family. All of these polymorphisms were within 1 Mb of the *COL2A1* variant. Oligonucleotide primers to amplify STRs and SNPs were designed in silico using Primer3Plus online software and checked for SNP exclusion by SNPCheck software (http://ngrl.manchester.ac.uk/SNPCheckV2/snpcheck.htm, accessed on 29 January 2020) and related databases (dbSNP, UCSC genome browser, http://genome.ucsc.edu, accessed on 29 January 2020). Moreover, the primers were designed to obtain amplicons with different lengths and were labelled with different dyes to be easily visualized after separation by capillary electrophoresis.

Informativity testing of each marker was checked on 100 ng genomic DNA from the couple and their parents (including the male partner’s affected father). Genomic DNA was isolated from EDTA anti-coagulated blood samples from the couple using a standard protocol (FlexiGene DNA Kit Qiagen, Courtabœuf, France) and from buccal swab samples from the couple’s parents (PrepIT•L2P, DNA Genotek Inc., Ottawa, ON, Canada). Eight informative polymorphic DNA sequences (4 STRs and 1 SNP on one side of the variant and 3 STRs on the other side) were selected for PGT-M purposes and tested in a multiplex PCR protocol (Table 2).

Pathogenic *COL2A1* variant testing was performed using a minisequencing approach [42].

Single lymphocytes from both of the couple were used as cell models for PCR development and validation of PGT-M. Lymphocytes were isolated from heparinized blood using UNI-SEP tubes according to the manufacturer’s protocol (NOVAmed, Jerusalem, Israel) and resuspended in the appropriate cell medium. Single lymphocytes were then isolated under an inverted microscope using a fine micropipette, lysed in 3 µL lysis buffer (200 mM KOH, 50 mM DTT) [43] for 10 min at 65 °C and then used immediately for PCR or stored at −20 °C until further processing.

PCR was performed in a final volume of 30 µL reaction mix using Qiagen Multiplex PCR Master Mix (Qiagen, Courtabœuf, France) and 0.08–1.16 µM each of the pairs of primers. PCR was performed on a GeneAmp PCR system 9700 (Applied Biosystems, Foster City, CA, USA) at 60 °C for 25 cycles (100 ng genomic DNA) or 40 cycles (single lymphocytes).

The PCR products were visualized by capillary electrophoresis on an ABI 3500 automated Genetic Analyser using the Genescan-500 LIZ Size Standard (Applied Biosystems, Courtabœuf, France). The results were analyzed by Gene Mapper 6.0 software (Applied Biosystems, Courtabœuf, France).

### 4.4. IVF-PGT-M

#### 4.4.1. Ovarian Stimulation

An antagonist protocol was scheduled using an estroprogestative pill (Minidril; Pfizer, France) for 20 days. The starting dose of gonadotropins (Fertistartkit; Genevrier, France; urofollitropin) was determined according to many clinical factors, such as the patient age, BMI, AMH, and AFC, and the results of the first attempt. The daily dose of 112.5 UI was initiated 5 days after pill cessation and remained constant for 9 days. The cycle was monitored from Day 6 by assessment of the plasma levels of LH progesterone and estradiol in conjunction with transvaginal ultrasound until hCG administration. On this cycle, the GnRH antagonist (Ganirelix—Orgalutran 0.25 mg, MSD, France) was introduced at Day 5 because a leading follicle achieved a diameter of 14 mm and the estradiol level reached 552 pg/mL. Ovulation was triggered with an hCG (Ovitrelle 250 μg; Serono, Switzerland) subcutaneous injection, administered because 3 follicles > 17 mm in diameter developed. Luteal support was achieved with vaginal progesterone 600 mg daily (Utrogestan; Laboratoires Besins International, SA, France) until the pregnancy test was performed.

#### 4.4.2. Ovarian Puncture and Gamete Fertilization

Transvaginal ultrasound-guided follicle retrieval was conducted 36 h after hCG administration. Antral follicles over 10 mm were aspirated using a single lumen 17 G needle connected to a vacuum aspiration system. Follicular fluids were collected at 37 °C until cumulus–oocyte complex collection. Oocytes were denuded mechanically and enzymatically with hyaluronidase (HYASE-10X™; Vitrolife). Only the mature oocytes (Metaphase II) were fertilized using an intracytoplasmic sperm injection on the day of ovarian puncture. Fertilization was assessed 16–18 h later, and the presence of 2 pronuclei (PN) signified successful fertilization (zygote stage). Embryo culture was performed with sequential media (G-1™ PLUS from Day 0 to Day 3 and G-2™ PLUS; Vitrolife).

#### 4.4.3. Embryo Biopsy and PGT-M

Developing embryos with 6 or more cells were biopsied on Day 3 (one blastomere/embryo). Blastomeres were individually processed as for lymphocytes (as described in the “PGT work-up” section).

### 4.5. Pregnancy

Ultrasound scans were performed with the Hera W10^®^, Samsung (Seoul, Korea).

### 4.6. Noninvasive Prenatal Diagnosis by Paternal Variant Exclusion

Noninvasive prenatal diagnosis was performed by droplet digital PCR as a personalized medicine service with a specific design of primers and probes as well as assay qualification for the *COL2A1* paternal variant c.2155C>T, as previously described [44]. Assays are composed of oligonucleotide primers and hydrolysis probes labelled with 5′-Fam or 5′-Hex for mutant and wild-type alleles, respectively. Upon receipt of the assay, the performance limit of the blank and the limit of detection of the assay were evaluated as well as linearity using serial dilutions of the paternal genomic DNA in maternal genomic DNA. A noninvasive prenatal test was performed on the maternal blood samples collected in BCT tubes. Paternal and maternal genomic DNA were amplified concomitantly as the positive and negative controls, respectively.

## 5. Conclusions

For the first time, we have provided molecular proof that a single blastocyst transfer can result in a monozygotic dichorionic diamniotic twin pregnancy. In PGT, it is necessary to assess the genetic status of dichorionic twins using prenatal diagnosis to exclude the risk from a potential concurrent spontaneous pregnancy. Further multicentric prospective studies using genetic testing are required to establish the rate of blastocyst splitting leading to dichorionic pregnancy, and thus to identify the risk factors associated with the safety of a twin pregnancy after single embryo transfer in PGT.

## Figures and Tables

**Table 1:** Clinical and biological parameters associated with dichorionic diamniotic twin pregnancy.

Parameter	Value
Maternal age (years)	34
Maternal Body Mass Index (kg/m^2^)	17.8
Antral follicle count (number)	26
Anti-mullerian hormone (ng/mL)	4.5
Paternal age (years)	35
Paternal Body Mass Index (kg/m^2^)	23.3
Sperm parameters	Normal
Gravidity and Parity	0/0
Number of days of stimulation	9
Total gonadotrophin dose (IU)	1012.5
Estradiol peak (pg/mL)	2189
Number of retrieved oocytes (Metaphase II)	17
Embryo biopsy	Day 3
Embryo transfer	Single blastocyst on Day 5
Embryo stage	4AA
Gestational weeks at delivery	36
Fetal weights (g)	2400/2090
Fetal gender	F/F
Zygosity	Monozygotic

**Table 2:** Sequences studied during the PGT-M cycle (location, sequences of the primers and distance to the pathogenic variant).

Markers/Variation (Location on Chromosome 12/GRCh38)	Primer Sequences	Distance to the *COL2A1* Pathogenic Variant (bp)	Amplicon Size (bp)
(TC)47.54 (47,541,504–47,542,343)	F: Hex-TGT GGT TTC TGT CTT GGG AGT R: TGC CAT ACT CCT TCT GTG TTC C	440,963	170–184
(TG)47.55 (47,557,867–47,558,698)	F: Hex-ATC TAT TTC AGG GCC CAG AGG R: ATC CTT GGA ACG ACA ATG GGT	424,604	213–227
D12S2175 (47,603,861–47,603,888)	F: Fam-AGC AAA TCA GTC TGT GTG CCT A R: TGC TTT GCA TAA TGC CTA TTT C	379,012	108–110
(GT)48.22 (47,833,117–47,833,147)	F: Fam-AAA AAC AGG CAG TGG AA R: GGA ACC CCA AAG CCT TAC TG	149,754	272–282
rs374243830 (47,982,664)	F: Hex-CCT GGG GAG GGA GGT AAG AG R: GGA AGG AAG AGG GGT TTG GG	222	101–102
c.2155C>T (g.47,982,886)	Multiplex PCR F: TGCTTCTCCCTGGACCTTCT R: GTTCATGGAGCCTGGGTAAC Mini sequencing F: TGCTTCTCCCTGGACCTTCT R: ACCCCTCTTCCTTCCCTTCC SnapShot MSF: TCTCCCGGTGCCCAGGGCCTCCAGGGTCCC	-	-
(TG)48.60 (48,215,576–48,215,618)	F: Hex-TTT TCT CTT CTG TGC CTT ATT GC R: TTC AGC TGT TCC ATG GCA TT	232,711	246–258
(TG)48.84 (48,447,213–48,447,251)	F: Rox-CTA CTG GCA CAG ATT CTA ACA GG R: TAT AGC CAC TTC CTG GCT TGT AG	464,346	254–258
(GT)49.25 (48,864,385–48,864,417)	F: Fam-GCT TTG CTC TGG GGA AGT AAA A R: GTG GGC AGT AAA ACA GGA CTT CT	881,515	146–149
